# Cdc7 overexpression is an independent prognostic marker and a potential therapeutic target in colorectal cancer

**DOI:** 10.1186/s13000-015-0360-7

**Published:** 2015-07-25

**Authors:** Nathaniel Melling, Johanna Muth, Ronald Simon, Carsten Bokemeyer, Luigi Terracciano, Guido Sauter, Jakob Robert Izbicki, Andreas Holger Marx

**Affiliations:** Institute of Pathology, University Medical Center Hamburg-Eppendorf, Martinistrasse 52, 20246 Hamburg, Germany; Department of Surgery, University Medical Center Hamburg-Eppendorf, 20246 Hamburg, Germany; Department of Oncology, Hematology, BMT with section Pneumology, Hubertus Wald Cancer Center, University Medical Center Hamburg-Eppendorf, 20246 Hamburg, Germany; Institute of Pathology, University Hospital Basel, Basel, Switzerland

**Keywords:** Biological markers, Cell cycle checkpoints, Colorectal neoplasms, Immunohistochemistry, Prognosis

## Abstract

**Background:**

Cdc7 is a widely expressed protein kinase implicated in cell division, cell cycle checkpoint mechanisms and cancer progression. Recently, it has been suggested as a target for anti-cancer therapy.

**Methods:**

To determine the relationship of Cdc7 protein expression with tumor phenotype, molecular features and prognosis, 1800 colorectal carcinomas were analyzed by immunohistochemistry on a tissue microarray.

**Results:**

Cdc7 expression was considered negative in 33.6 %, weak in 57.2 % and strong in 9.2 % of 1711 interpretable CRCs. Loss of Cdc7 expression was significantly associated with high tumor stage (*p* < 0.0001) and high tumor grade (*p* = 0.0077), but was unrelated to the nodal status (*p* = 0.5957). Moreover, a link between Cdc7 expression and the tubular histological tumor type was seen (*p* < 0.0001). p53 and Cdc7 expression were significantly linked to each other (*p* = 0.0013). In a multivariate survival analysis, strong Cdc7 expression of CRC was an independent marker of improved patient survival (*p* = 0.0031).

**Conclusion:**

Our data show that Cdc7 is highly expressed in CRC and a potential therapeutic target in a subset of cancers with high p53 expression. Moreover, our findings strongly argue for a clinical utility of Cdc7 immunostaining as an independent prognostic biomarker in colorectal cancer enabling to select patients for adjuvant treatment.

## Background

Colorectal cancer (CRC) is the fourth most common malignant disease with over 1 million novel cases and over 500.000 deaths each year worldwide [1].

Although recent advances in the management of the disease have improved outcomes, CRC remains the second leading cause of cancer-related death in Western countries [1]. In advanced metastatic colorectal cancer (mCRC), surgery alone is not curative and therefore adjuvant chemotherapy is needed. There is much promise in targeted anti-cancer therapies. Encouraged by the success of targeted therapies directed against key molecules of cell growth like the HER2, EGFR, or KIT tyrosine kinase receptors, researchers have extended their survey for promising candidate targets to genes that participate in the control of cell division and replication. Such genes represent the most downstream effectors of growth signaling pathways and therefore it is believed that their inhibition will be effective in a broad range of different tumor types characterized by rapid cell proliferation.

The cell division cycle 7- (Cdc7) related protein kinase is an enzyme that is essential for DNA replication in human cells [2, 3]. Overexpression of Cdc7 and its protein regulator Dbf4 has been reported in many human tumors [4], including ovarian cancer [5], melanoma [6, 7], diffuse large B-cell lymphoma [8], oral squamous cell carcinoma [9] and breast cancer [10]. Cdc7 and Dbf4 overexpression are reported to cause cell-cycle arrest in S phase and it has been hypothesized that increased Cdc7 activity may aid recovery or repair of stalled replication forks to enhance survival of tumor cells [11]. Therefore, it can be assumed that alterations in Cdc7/Dbf4 protein activity during tumorigenesis may have important consequences for tumor cell survival, underlining the potential of Cdc7 as an anticancer target. High expression of Cdc7 protein correlates with poor prognosis in patients with diffuse large B-cell lymphoma and is a marker of resistance to DNA-damaging agents in oral squamous cell carcinoma [9, 8]. Preclinical data show that Cdc7 is a novel and promising target for tumor-cell killing, as has been shown with different inhibitors [12–15]. Little is known about the prevalence and significance of Cdc7 in colorectal cancer. To study the potential role of Cdc7 in CRC a tissue microarray containing 1.800 tumor samples with clinical follow-up data was analyzed. Our data suggest that Cdc7 overexpression may point out a small but significant subset of immunohistochemically p53-positive CRC that could benefit from anti-Cdc7 treatment.

## Methods

### Patients and tissue microarray (TMA) construction

Two different TMAs with a total of 1800 CRC samples were included in this study. The first TMA was manufactured from resection specimens of 1420 CRC patients at the Institute of Pathology of the University Hospital of Basel. None of the patients received preoperative neo-adjuvant or adjuvant therapy. Raw survival data were obtained from the responsible physicians for all of the 1420 patients. The median follow up time was 46 months (range 1–152 months). The second TMA included samples from 380 CRC patients, whose tumor resection specimens were examined at the Institute of Pathology of the University Medical Center Hamburg-Eppendorf. Also for this TMA, raw survival data were available for all of the 380 patients with a median follow up period of 36 months (range 1–179 months). TMA construction was as described [16]. In brief, hematoxylin and eosin-stained sections were made from each block to define representative tumor regions. Tissue cylinders with a diameter of 0.6 mm were then punched from tumor areas of each “donor” tissue block using a home-made semi-automated precision instrument and brought into empty recipient paraffin blocks. Four μm sections of the resulting TMA blocks were transferred to an adhesive coated slide system (Instrumedics Inc., Hackensack, New Jersey). Patient information and clinical data as age, sex, localization and type of the tumor, pTNM-stage and carcinoma grade were retrospectively retrieved from clinical and pathological databases (Table 1). All tumors were re-classified by two pathologists (LT, AM). Follow-up data were obtained from local cancer register boards or via attending physicians. For statistical analyses, tumor localizations were grouped as follows: right-sided cancer (cecum, ascending colon), cancer of the transverse colon, cancer of the left-sided colon (descending colon, sigmoid colon) and rectum.

**Table 1:** Clinical and pathological features of colorectal cancers

Clinical/pathological features		n available
Gender	Female	858
	Male	853
Age	Mean: 69 (29 – 96)	
Tumor grade	G1	29
	G2	1505
	G3	177
Tumor stage	pT1	75
	pT2	270
	pT3	1104
	pT4	262
Nodal status	pN0	889
	pN1	458
	pN2	364
Tumor type	Tubular carcinoma	1644
	Mucinous carcinoma	59
	Others	8
Tumor localization	Right colon	414
	Transverse colon	149
	Left colon	514
	Rectum	637
Total number of patients		1711

The utilization of tissues and clinical data was according to the Hamburger Krankenhaus Gesetz (§12 HmbKHG) and approved by our local Ethical Committee.

### Cdc7 immunohistochemistry

Standard indirect immunoperoxidase procedures were used for the detection of Cdc7 (abcam, clone SPM171, dilution 1:150). Sections were heated in an autoclave at 121 °C for 10 min in citrate buffer (pH 9.0). Diaminobenzidine was used as a chromogen and sections were counterstained with Mayer’s hematoxylin. Immunostaining was typically nuclear. For tumor tissue the percentage of positive cells was estimated and the staining intensity was recorded as 1+, 2+ or 3+. For statistical analyses, the staining results were categorized into three groups as previously described [17]. Tumors without any staining were considered “negative”. Tumors showing at least weak Cdc7 staining were considered “positive”. Tumors with 1+ or 2+ positivity in up to 50 % or 3+ positivity in up to 20 % of cells were considered “weakly positive”. Tumors with 2+ staining in >50 % or 3+ staining in >20 % of cells were considered “strongly positive”. The molecular database attached to this TMA contained results on p53 expression in 1800 cancers.

### Statistics

Statistical calculations were performed with JMP® 10.0.2 software (2012 SAS Institute Inc., NC, USA). Contingency tables and the chi^2^-test were performed to search for associations between molecular parameters and tumor phenotype. Survival curves were calculated according to Kaplan-Meier. The Log-Rank test was applied to detect significant survival differences between groups. Cox proportional hazards regression analysis was performed to test the statistical independence and significance between pathological and clinical variables.

## Results

### Technical issues

A total of 1711 (95.1 %) of tumor samples were interpretable in our TMA analysis (Table 1). Reasons for non-informative cases (289 spots; 4.9 %) included lack of tissue samples or absence of unequivocal cancer tissue in the TMA spot.

### Cdc7 expression in colorectal cancer

**Fig. 1:**
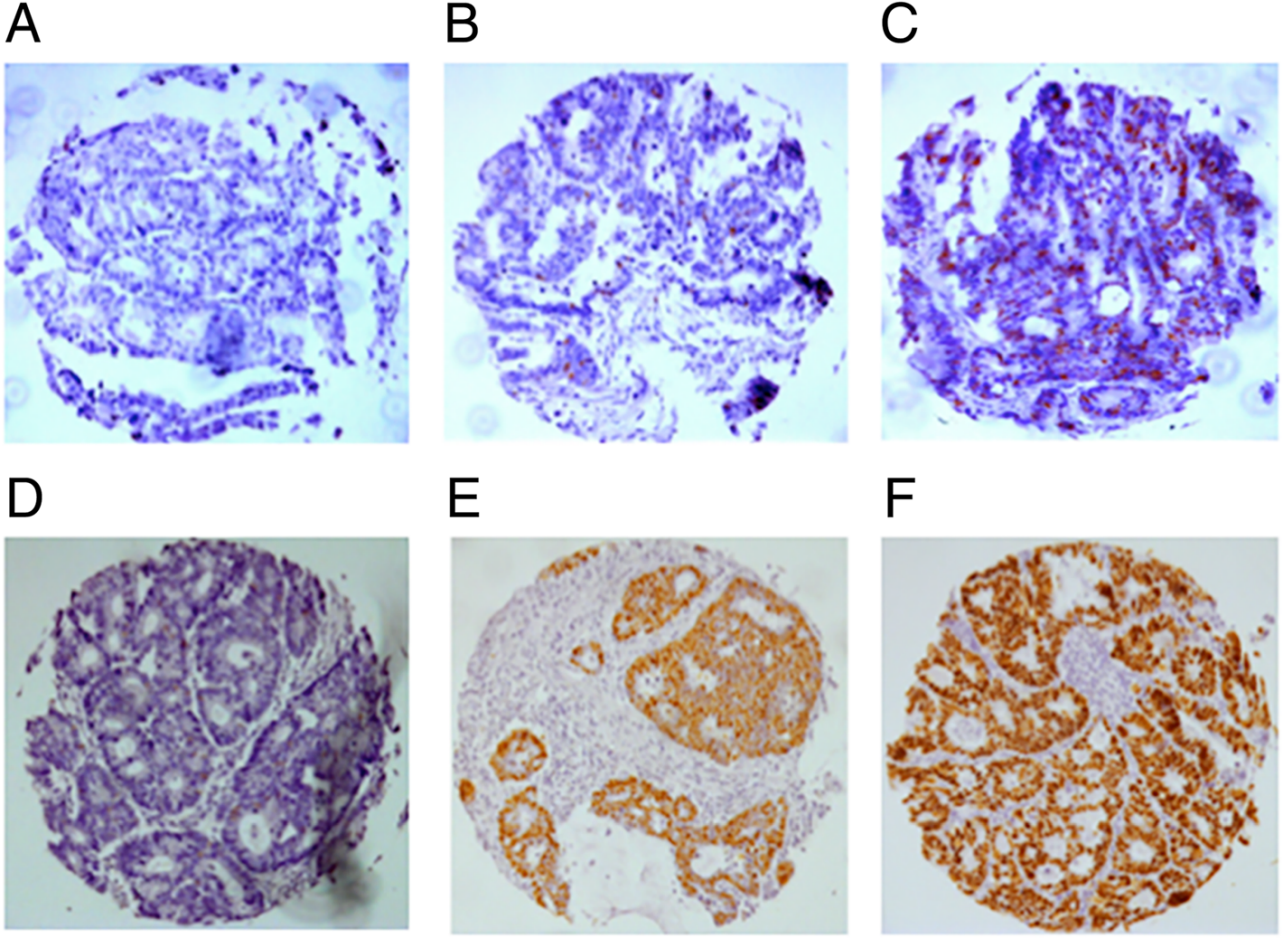
Representative images of immunohistochemical Cdc7 and p53 expression in colorectal cancer: **a** Cdc7 negative; **b** Cdc7 low and **c** Cdc7 high expression, **d** p53 negative; **e** p53 low and **f** p53 high expression, magnification 50 x each

Cdc7 expression was considered negative in 33.6 %, weak in 57.2 % and strong in 9.2 % of 1711 interpretable CRCs. Representative images of Cdc7 IHC are given in Fig. 1a - c. Loss of Cdc7 expression was significantly associated with high tumor stage (*p* < 0.0001) and high tumor grade (*p* = 0.0077), but was unrelated to the nodal status (*p* = 0.5957, Table 2). Moreover, a link between Cdc7 expression and the tubular histological tumor type was seen (Table 2, *p* < 0.0001).

**Table 2:** Cdc7 IHC, clinico-pathological and molecular parameters in CRC

		Cdc7			
Parameter		Negative (%)	Weak (%)	Strong (%)	*p*-value
All cancers	1711	33.6	57.2	9.2	
Tumor stage					
pT1	75	25.3	53.3	21.4	
pT2	270	24.1	64.8	11.1	
pT3	1104	34.6	57.5	7.9	
pT4	262	41.6	48.9	9.5	<0.0001
Nodal status					
pN0	889	32.2	58.7	9.1	
pN1	458	36.2	55.0	8.8	
pN2	364	33.8	56.0	10.2	0.5957
Grading					
G1	29	34.5	37.9	27.6	
G2	1505	32.8	58.3	8.9	
G3	177	40.7	50.3	9.0	0.0077
Tumor localization					
Right	414	37.2	53.9	8.9	
Transverse	149	37.6	54.4	8.0	
Left	514	31.6	60.2	8.2	
Rectum	637	30.8	58.3	10.9	0.2093
Histological type					
Tubular	1644	33.5	57.6	8.9	
Mucinous	59	37.3	50.8	11.9	<0.0001
p53					
Negative	530	36.6	53.6	9.8	
Positive	376	25.3	63.0	11.7	0.0013

### Association with tumor localization

Cdc7 expression levels were not related to tumor localization (Table 2, *p* = 0.2093).

### Association with p53 expression

p53 expression was significantly linked to Cdc7 expression (Table 2, *p* = 0.0013). Representative images of p53 IHC are given in Fig. 1d - f.

### Survival analysis

**Fig. 2:**
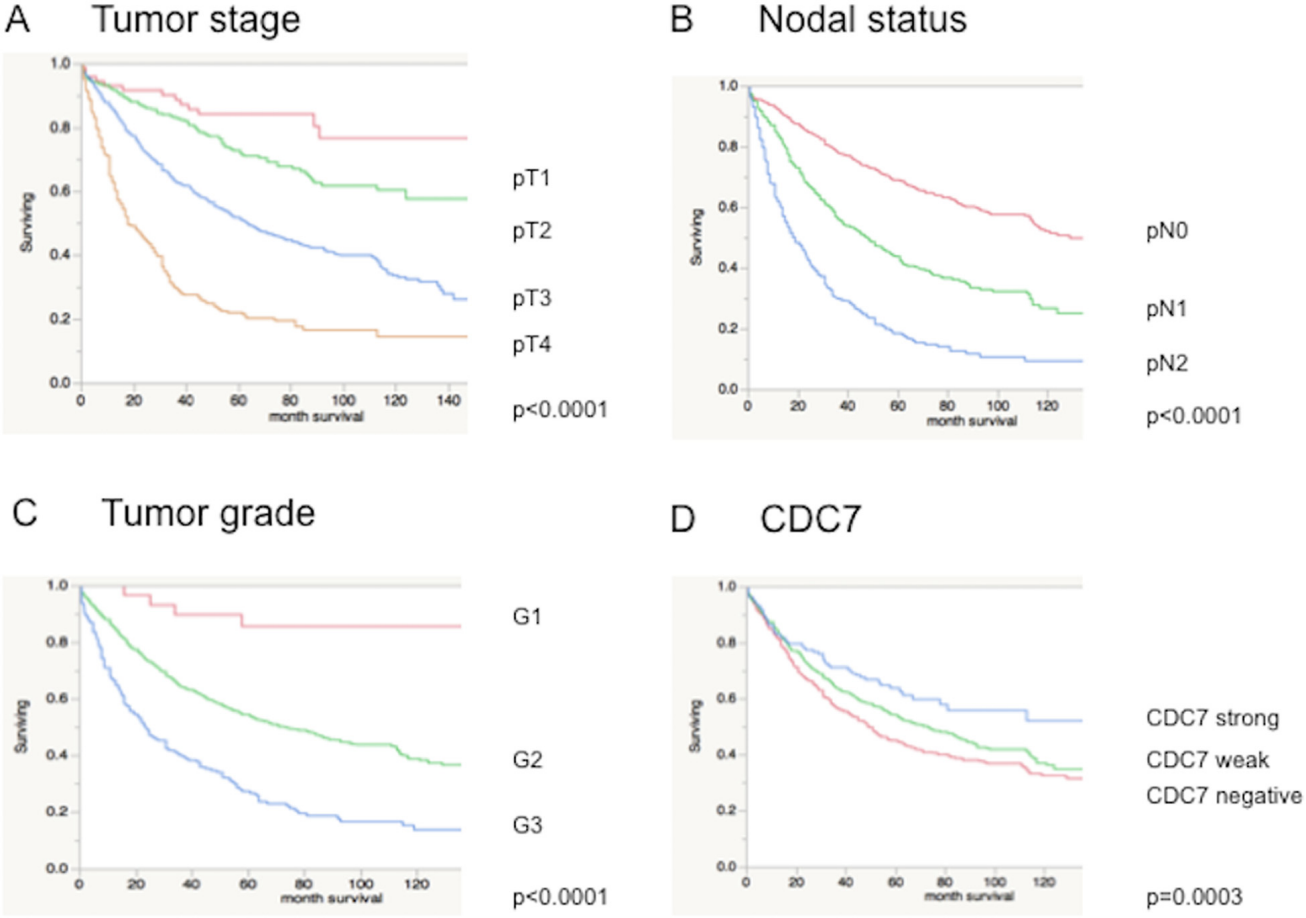
Association between survival and **a** Tumor stage, *p* < 0.0001; **b** Nodal status, *p* < 0.0001; **c** Tumor grade, *p* < 0.0001 and **d** Cdc7 expression (Cdc7 strong-blue, Cdc7 weak-green, Cdc7-negative-red); *p* = 0.0003

As expected, high tumor grade and stage as well as advanced nodal status were associated with poor patient survival (Fig. 2a-c; *p* < 0.0001 each). Strong Cdc7 expression in CRC was significantly related to improved patient survival compared to cases with loss of Cdc7 expression (Fig. 2d; *p* = 0.0003). Immunohistochemical p53 status in the CRC did not show any impact on survival (*p* = 0.3735; data not shown).

### Multivariate analysis

In a multivariate analysis including all parameters that were significantly associated with survival on univariate analysis (pT, pN, tumor grade Fig. 2a-c, and tumor localization; data not shown), Cdc7 expression retained significance as an independent prognostic marker (Table 3, *p* = 0.0031).

**Table 3:** Multivariate analysis of Cdc7 in colorectal cancer

*p*-value					
n cases	pT	pN	Grading	Tumor localization	Cdc7
1711	<0.0001	<0.0001	0.0008	0.8200	0.0031

## Discussion

The results of this study show that in CRC strong Cdc7 expression is an independent favorable prognostic marker and significantly linked to immunohistochemical p53 positivity. Cdc7 expression was found in 66.4 % of all interpretable CRCs, and it was typically described as weak in normal tissue. Only few reports on results of Cdc7 expression in CRC exist. Chen et al. reported a significantly higher mRNA and protein expression of Cdc7 in 39 colorectal cancers as compared to tumor-adjacent normal colorectal tissue [18] and colorectal cancer is among the cancer types that shows the strongest up regulation in Cdc7 expression as determined from data obtained by mining Oncomine and Gene Expression Omnibus databases. Bonte et al. examined Cdc7 expression in an array of human malignancies, including colon cancer where they found low Cdc7 expression in 2 and high Cdc7 expression in 8 of 10 cases [4].

The percentage of CRC cases with "strong" Cdc7 staining in our study is 9.2 %. This may be attributable to a mismatch between mRNA and protein levels, but it is more likely caused by immunohistochemistry conditions. It is well known that varying antibody dilutions lead to significant changes in the rate of positive cases [19]. This is all the more expected in the case of ubiquitously expressed proteins, such as Cdc7.

Various functional studies had previously demonstrated that Cdc7 is essential for cell proliferation [20, 21]. The strong association between Cdc7 and p53 overexpression found in our study was therefore expected, although p-values may appear stronger than the absolute values imply, due to the high caseload (e.g. p53 positive vs p53 negative 11.7 % vs 9.8 %; *p* = 0.0013). A previous study has shown that a p53-dependent pathway in normal fibroblasts actively prevents progression through a lethal S phase in the absence of sufficient Cdc7 kinase. In contrast, Cdc7 and p53-depleted tumor cells do not elicit a robust checkpoint response. Therefore, it has been suggested that p53 is required for cell viability in Cdc7 deficient cells [22]. Moreover, it has been reported that ATR-dependent activation of p38 MAP kinase is responsible for apoptotic cell death in Cdc7-depleted cells [23]. In CRC, up to 42 % of the cases show p53-mutations with a strong inverse correlation between p53 alterations and MSI [24]. Thus, we hypothesize that Cdc7 inhibition may serve as a new therapeutical approach in a subgroup of p53-deficient CRC, which are predominately localized in the left colon [24].

In our study, Cdc7 overexpression proved to be an independent marker for good prognosis in CRC. This finding is in contrast to studies on other cancer types where Cdc7 overexpression is linked with worse prognosis. For example, Cheng et al. reported in their study on oral squamous cell carcinoma (OSCC) that Cdc7 overexpression is an unfavorable prognostic marker and suggested Cdc7 overexpression contributing to the resistance to DNA-damaging agents [9].

In another recent study of our group on more than 2.100 breast cancers, Cdc7 expression was seen in 57 % of the cases. Comparable to what was found in CRC, in breast cancer high levels of Cdc7 expression were also significantly related to high tumor grade, high Ki67 expression and p53 overexpression. Although the IHC scoring system was slightly different in this breast cancer study (4 categories vs 3 categories as described earlier) [17], the percentages of Cdc7 negative, weakly and strongly positive breast cancers were similar to our findings in CRC [10].

However, when comparing Cdc7 expression and tumor grade and stage, results in both studies opposed each other. Whereas in CRC low levels of Cdc7 expression were significantly associated with high tumor grade and stage, in breast cancer high levels of Cdc7 expression were linked with higher tumor grade and stage [10]. The reason for this discrepancy is unknown but description of variable biological roles of one biomarker in different tumor entities is not uncommon. For example, AQP5 expression is associated with poor prognosis in breast cancer [25–27], hepatocellular carcinoma [28], colorectal carcinoma [29, 30], chronic myelogenous leukemia [31], lung cancer [32, 33], ovarian cancer [34, 35], cervical cancer [36] and oral squamous cell carcinoma [37], but to better outcome in gallbladder [38] and biliary tract cancer [39].

Furthermore, some biomarkers, such as GSK3ß, are known to play both tumor suppressive and promoting roles depending on the microenvironment It has, thus, been suggested that GSK3ß function may switch between tumor promotion and suppression depending on cell properties and the stage of tumorigenesis [40].

Therefore, varying oncogenic properties of Cdc7 might also be attributable to differing molecular backgrounds in various cancer types.

Of note, Cdc7 has been shown to represent a potent and highly specific anticancer target in p53-mutant, Her2-overexpressing and triple-negative breast cancers [41]. Based on these findings, a number of different Cdc7 kinase inhibitors which showed anti-tumor activity have been developed recently [12–14]. Our data show that Cdc7 is expressed in the majority of colorectal cancers and therefore could serve as a potential therapy target.

It is well known that immunostaining of p53 can serve as a surrogate marker for p53 mutations in cancer [42]. Thus, CRC, which show p53 overexpression are likely to harbor p53 mutations and therefore may be susceptible to anti-Cdc7 therapy [22].

However, experimental and clinical studies examining the efficacy of Cdc7 kinase inhibitors in CRC are still needed as well as data on Dbf4 protein expression, since colorectal cancers have been shown to have strong levels of Dbf4 mRNA expression and Dbf4 expression levels were uniformly high among most colorectal tumors (Bonte et al.). This data could well be recruited by a TMA approach similar to ours.

## Conclusion

In summary, our data show that Cdc7 is highly expressed in CRC and may represent a potential therapeutic target in a subset of cancers with high p53 expression.

Finally, our findings strongly argue for a clinical utility of Cdc7 immunostaining as an independent prognostic biomarker in colorectal cancer enabling to select patients for adjuvant treatment.
